# The Foreign Journals: Surgery

**Published:** 1841-04

**Authors:** 


### SURGERY.

### *Partial Injury of one of the Halves of the Spinal Marrow.* By M. Begin.

L., a robust old soldier, aged fifty-nine, received a wound from a sharp-pointed cutting instrument in the back of the neck. He fell immediately, and being unable to raise himself, was obliged to be carried to an adjacent house. He said he had been knocked down by some heavy weapon, and that he had fallen not in consequence of the wound in the neck, which he thought insignificant, but from the violent stunning which he felt at the instant he was struck. He fell *backwards* and on his right side, and there was a slight bruise on the right elbow, but no other sign of external violence, although he attributed the impossibility of moving his right limbs to the shock and the bruises which he had received in his fall, or more still to the blows which he affirmed had been given him while on the ground.

The wound was united by plaster, and the patient, regarding his injury as a slight matter, refused to be bled. On the next day he was taken to the Val-de-Grace, where M. Begin first saw him, in the evening. He then said that he felt nothing but a little numbness of the right side. His pulse was slightly excited; the wound had healed, and he would not be bled. Cold was applied to the head and neck, and warmth to the feet, and antiphlogistic diet was ordered. He passed the night quietly. Next day, on examining the united wound more closely, it was found to be in a transverse direction, half an inch long, at the level of the fifth cervical vertebra, and nearly an inch from its spinous process. Its angles were nearly equally acute, rendering it probable that it had been made with a double-edged instrument; there was neither ecchymosis, nor swelling, nor heat, nor pain, nor hardness about it; the movements of the head and neck were perfectly free, and unaccompanied by pain, even when carried to a considerable extent. The patient felt a kind of weight in his right arm, and had creeping sensations in the hand; with a little difficulty, however, he could raise the arm, and move the fore-arm; but the fingers half-flexed could neither be opened nor closed completely. The right leg was absolutely motionless. A vague pain was felt along the right side of the chest, and there was perfect sensibility in every part of the body. The mind was clear: there was slight fever. The patient was bled freely both from the arm and locally; stimulants were applied to the feet, and cold to the head, and strict abstinence was observed.

On the third day after the accident there was a slight power of moving the right great toe. But there was much more fever, and the patient was again freely bled. On the fourth day, after a very quiet night, he was a good deal better, but at night he became feverish, excited, and delirious. This condition was somewhat relieved by leeches; but next day he had a violent shivering-fit, followed by incomplete reaction; at night he had low delirium, with an unequal pulse, difficult, hurried respiration, and hiccup, and with these increasing he died early in the morning of the sixth day after receiving the wound.

At the examination of the body, the wound externally was scarcely discernible, but more deeply its course became more and more evident by the effusion of blood in the adjacent tissues. In the middle of the right lamina of the sixth cervical vertebra there was a fragment of a knife, which projected backwards for about a line, and had its back directed towards the median line. Having removed all the cervical portion of the vertebral column, and cleaned its fore-part, the point of the same portion of knife was found projecting about one tenth of an inch between the sixth and seventh vertebræ. It had broken the upper edge of the body of the latter, and had stuck into, but not passed through, the posterior wall of the pharynx. The left side of the column was cut away, and the vertebral canal being thus opened, the medulla spinalis was found surrounded by pus mixed with the spinal fluid, and its surface was softened above and below the wound. It had been struck by the back or rather by the blunt edge of the blade of the knife, and the cut extended obliquely on the right side, from the level of the origin of the posterior roots of the nerves to the anterior median furrow. The posterior column was uninjured from the line of origin of the posterior roots to the posterior median furrow, while the anterior column was divided from the same point to the groove occupied by the anterior spinal artery.

The portion of the weapon that remained in the wound was an inch and three quarters long, and three fifths of an inch broad at its base; it was part of a short and very sharp dagger-knife.

The shock that must have accompanied the violent penetration of the arch of the sixth vertebra, and the intervertebral cartilage, as well as the part of the body of the seventh vertebra, perfectly explains the sensation of bruising and the stunning which the patient felt at the instant of receiving the wound. Neither during life nor after death was there any erection of the penis.

[This case is interesting in every way, but especially in its obvious bearing on the functions of the columns of the spinal marrow, and the nerves respectively connected with them.]

*Bulletin de l'Académie de Médecine. Dec.* 15, 1840.

---

*On the Removal of Foreign Bodies in the Joints by means of Subcutaneous Incisions.* By Dr. Goyrand, of Aix.

This new method of operating was suggested by the fact, that incisions made into joints through such small external openings that the air cannot obtain admittance to the cavity, are never productive of that severity of inflammation which follows wounds with wide external apertures, and which, in the operations hitherto performed for this disease, has so often terminated fatally. The proceeding recommended by the author consists in pushing the loose body (if in the knee-joint,) into the synovial pouch above and to the outer side of the patella, beneath the vastus externus muscle, and while an assistant holds it fixed there, passing a narrow knife through the skin at some distance above the joint, and through all the intermediate tissues down to the foreign body. Without enlarging the opening in the skin, the synovial membrane and adjacent tissues over the loose substance are now to be freely divided, till, by the pressure on the latter, it slips out of the joint through the wound, and lodges itself in the subcutaneous cellular tissue, or in some of the other tissues between the skin and the joint. After this the patient must remain at rest for several days, (the small external aperture being merely covered by sticking-plaster,) till all chance of inflammation occurring has passed away. The foreign body dislodged from the interior of the joint, will form a cyst for itself, and remain in its new position without producing any annoyance; but, if it should be deemed necessary, it may easily be removed by a single incision through the skin over it, which will no longer be likely to excite any inflammation of the joint itself.

The author details a case in which this method of operating was adopted with perfect success. Two loose cartilages were dislodged, and pushed into the tissues adjacent to the joint. One was subsequently removed by an incision through the skin; the other was allowed to remain, and it produced no inconvenience whatever. Through the whole course of the treatment the patient had not a bad symptom, although less precautions were adopted than are usual in in this description of cases.

[We understand that Mr. Syme, of Edinburgh, performed this operation, and with complete success, without previous knowledge of M. Goyrand's proceeding.]

*Annales de la Chirurgie Française et Etrangère. Janvier,* 1841.

---

*Successful performance of Tracheotomy in a case of Croup.* By M. Saussier.

The case occurred in the hospital of la Pitié, in a boy two years old, who had been operated on for hare-lip, by M. Sanson. The first croupy symptoms appeared ten days after the operation, and after the unsuccessful employment of leeches and emetics the child seeming to be in imminent danger of suffocation the operation was performed. White patches had formed upon the tonsils and arches of the palate, afterwards on the tongue and internal surface of the cheeks, and deglutition had become impossible.

The operation was followed by instant relief. M. Saussier cleaned out the trachea with a sponge, which he introduced several times, and upon which he withdrew a transparent pellicle, about two centimetres broad. Ten minutes afterwards, the canula was introduced, and the child almost immediately fell asleep.

During the three following days, eleven distinct, thick, yellow, false membranes were either expelled naturally by the patient, or withdrawn from the trachea by means of the sponge. The trachea was cauterized four times with a solution of sixty centigrammes of nitrate of silver in four grammes of water. The canula was changed thrice.

The canula was finally removed on the thirteenth day, and the teasing cough and frequent expectoration of thick mucosities which had continued till then, immediately ceased. On the sixteenth day the wound was healed, and at the end of August the child was discharged, having been operated on on the 3d of June.

*L'Expérience. Dec.* 3, 1840.

---

*On the Efficacy of Chloride of Zinc in the Treatment of Acute and Chronic Gonorrhœa.* By M. GAUDRIOT.

AFTER a large number of observations the author of this memoir concludes that the chloride of zinc, properly diluted, has a remarkable power in curing simple gonorrhœa of the urethra and vagina, also in dilating the urethra, and thereby ameliorating strictures. He believes that it is always by an inflammation *sui generis* that the discharge is cured, and that injections of the chloride of zinc determine a series of phenomena not produced by other caustics. It is only necessary to use a few drops of injection, as the extremity of the urethra is the only part to which it should be applied. To combat vaginal gonorrhœa in women M. Gaudriot proposes a new proceeding, which consists in the use of solid vaginal suppositories, composed of a paste made to melt easily, and containing a certain quantity of liquid chloride of zinc and sulphate of morphine.

In men the author employs the following formula:

Liquid chloride of zinc, 24 to 36 drops.
Distilled water, four ounces.
Agitate and filter through paper.

A small quantity of this solution should be injected about an inch along the urethra, two or three times a day.

The vaginal suppository which he employs is formed of

Liquid chloride of zinc, 5 drops.
Sulphate of morphine, half a grain.
Mix with three drachms of the following paste!
Mucilage of gum tragacanth, 6 parts.
Powdered sugar . . . . 3 „
Starch powder . . . . 9 „

To cure radically a gonorrhœa in man, it will ordinary suffice to apply two injections a day for two or three days. The first injections are almost always followed by more or less swelling of the glans penis, but this does not prevent their continuance.

In women four or six suppositories, one being introduced every day, or every second day, suffice to obtain a cure. The first introductions frequently occasion a swelling with more or less heat of the vulva, but these phenomena are soon completely dissipated. Emollient baths have in most of the cases been employed for this purpose.

*Journal des Connaissances Médicales Pratiques et de Pharmacologie. Sept.* 1840.

### *On Ligature of the common Carotid Artery, and Experiments on the Ligature of both Carotids in Animals.* By M. Jobert, of Lamballe.

The author addressed a memoir to the Royal Academy of Medicine, containing a case where he tied the common carotid to cure an erectile tumour of the orbit, and also the result of his experiments on living animals, to determine the influence that ligature of both carotids would have upon them.

Ligature of the common carotid on either side does not stop the course of the blood through the ramifications of the tied artery; how then can it effect the cure of erectile tumours of the face and head? M. Jobert replies, 1st, by the sudden subtraction of a large quantity of blood from the tumour; 2, by giving an obstacle to the transmission of the impulse of the heart, in its full energy towards the tumour. He has convinced himself by his experiments, that beyond the ligature, the blood runs in a continual jet, waving, and without jerk (*saccade*). His conclusions are, 1st, that erectile tumours of the orbit, destroy the bones after the manner of aneurisms; 2d, they have the characters of aneurismal tumours, and are cured by ligature of the common carotid of the same side; 3d, the cure is not owing to obliteration of the artery beyond the ligature, but to diminished impulse of the column of blood arriving in the tumour; 4th, the vertebral arteries suffice for the cerebral circulation after ligature of the carotids; 5th, dogs, sheep, and rabits do not experience ill effects after this operation; 6th, horses on the contrary do not survive it, dying with pulmonary apoplexies; 7th, bloodlettings before or after the ligature, diminish the intensity of the pulmonary lesions; 8th, perhaps, in man, the loss of a certain quantity of blood after the operation, would have good effect.

*Revue Médicale. Septembre,* 1840.

MM. Bérard, Gimelle, and Larrey, who were appointed as a committee to examine the memoir of M. Jobert, consider it of importance; 1st, as adding two cases of cure of erectile tumours, one in the orbit by ligature of this carotid, to four other cases previously recorded; 2d, as the experiments prove the harmlessness of ligature of both carotids, in those animals whose vertebral arteries enter the cranium of a caliber sufficiently large to keep up the cerebral circulation and avoid pulmonary congestion; 3d, as it proves that the brain and the organs of the senses preserve their functions entire after this ligature; 4th, as it places beyond doubt, that animals who from their anatomical construction survive the ligature of the common carotid arteries, are not affected with lesions of the organs of the senses as described by authors; 5th, as these experiments and their results will exercise a great influence on the surgical therapeutics of diseases, for the cure of which ligature of the common carotids might be proposed.

*Bulletin de l'Académie Royale de Médecine.* 15 *et* 30 *Octobre,* 1840.

---

### *Stone in the Tonsils.* By Dr. Wiesner, of Heydekrug.

A man seventy-six years old had for seven years often suffered from inflammation and swelling of the right tonsil. The affection was palliated by the means that were employed, but it often appeared again at different times after being heated and chilled, and at last a hardness of the tonsil remained, which gradually increased, becoming uneven and knotty to the touch, and, on the slightest contact causing severe pain, and rendering it difficult to swallow.

One morning, full seven years after the first occurrence of the angina tonsillaris, the patient on waking had, on attempting to eject some mucus that was collected in his mouth, felt a loose body in it, and drew out with his fingers a porous stone, like a urinary calculus and as big as two hazel-nuts. No bleeding occurred, and the opening in the tonsil soon closed after using an astringent gargle; at a later period, however, an inflammatory swelling of the left tonsil took place, and presented every appearance of a stone being in process of formation there.

*Casper's Wochenschrift. August* 15, 1840.

*Radical Cure of Varicocele by means of Invagination and Shortening of the Scrotum.* By Dr. LEHMANN.

THE mode of operating here recommended is very similar to that which the author has in several cases adopted successfully for the radical cure of hernia. A portion of the relaxed and elongated scrotum is to be pushed up on the fore-finger of the left hand, and *invaginated* into the part above it, till the finger reaches the external abdominal ring. Holding the parts in this position, a broad curved needle, with a double thread passed through an eye near its point, is to be carried along the left finger, through the bottom of the inverted portion of the scrotum, and to be made to penetrate it and the integuments immediately over the external ring. The thread is then to be removed from the eye, and the needle drawn back and again carried through the inverted portion of the scrotum and the integuments at a distance of about half an inch from the parts previously penetrated. The threads passed through the two apertures being now drawn, the invaginated portion of scrotum may be pulled up to any desired height; in general it must be drawn very nearly to the external ring; for in the subsequent progress of the cure the relaxation of the parts is so great that the folds and wrinkles thus produced are almost entirely obliterated. The threads then having been drawn must be knotted, and the parts thus tied up must be left for eight or nine days, under a soothing and cooling regimen, by which time adhesion will have taken place between them at the cut edges, and, by the excoriation which the discharge produces between the opposed surfaces of the inverted portion of the scrotum and that into which it is pushed.

The author relates six cases in which his mode of operating was adopted with success; but in none of them had sufficient time elapsed before the publication of the paper to enable one to say whether this measure was likely to be more permanently beneficial than the others which have been proposed for curing varicocele by bringing on the same change in the scrotum. Of all these the simplest and, as far as we have seen, the most successful, is that recommended by Mr. Wormald. (See Brit. and For. Med. Rev., vol. VI., p. 274.)

*Med. Zeitung. Dec.* 2, 1840.

---

*Mode of Action of Cubebs and Copaiba.* BY M. RICORD.

AT the sitting of the Royal Academy of Medicine on the 8th of September last, M. Ricord showed a design of a case of accidental hypospadias, resulting from a urinary abcess. The patient affected with this infirmity having contracted a gonorrhœa, it gave rise to some curious observations. The discharge first showed itself in the vesical portion of the urethra afterwards the part situated before the solution of continuity was invaded in its turn. Treated by copaiba, the vesical portion was soon cured, but the disease continued in the other part, and afterwards communicated it to the portion already cured. Cubebs was administered, and the discharge again ceased in the posterior portion of the canal. These facts show, according to M. Ricord, that cubebs and copaiba cure syphilitic discharges by the principles or properties which they communicate to the urine, and of which the urethra receives the influence by the passage of that fluid.

*Archives Générales de Médecine. Octobre,* 18 40.

---

*Practical Considerations on the Treatment of White Swellings.* By Dr. FORGET.

THE only novelty in this paper is an account of the employment of the muriate of baryta (chloride of barium,) in cases of chronic arthritic disease, as used in the hospital of La Pitié. The author considers it to be a most useful medicine, both in the acute and chronic state of white swellings, especially in scrofulous patients, and believes that in some cases it has alone sufficed to effect a cure, while usually great amendment has followed its use, though the cure may not have been complete. The usual dose has been six decigrammes, continued for a month. The circulation is very generally lowered, the pulse being depressed from sixty or eighty to forty or even twenty-five [?] in the minute. Local bleeding and compression have been advantageously combined with the employment of the baryta.

*Bulletin Général de Thérapeutique.* 15 *et* 30 *Septembre,* 1840.

---

*Extraction of a Prune-stone, which had remained eleven days in the Air-passages.* By M. Bonnet, Surgeon to the Hôtel Dieu, at Lyons.

The symptoms in this case were convulsive cough, with *complete* remissions. The cough increased in severity and continuance; and eleven days after the accident death appeared so imminent that the parents objected to an operation as useless. The chest was clear on percussion, but there was such an absence of the respiratory murmur as could only be attributed to some mechanical obstacle to the entrance of air into the lung. The signs of elevation and depression of the foreign body could not be recognized. The operation of tracheotomy was performed in the usual manner, but six ligatures had to be applied to vessels of the thyroid gland. The stone was removed by forceps, and complete recovery followed. The child was eleven years of age.

*Bulletin Général de Thérapeutique.* 15 *et* 30 *Septembre,* 1840.

